# Threshold of 25(OH)D and consequently adjusted parathyroid hormone reference intervals: data mining for relationship between vitamin D and parathyroid hormone

**DOI:** 10.1007/s40618-023-02057-9

**Published:** 2023-03-15

**Authors:** M. Gong, K. Wang, H. Sun, K. Wang, Y. Zhou, Y. Cong, X. Deng, Y. Mao

**Affiliations:** 1 Department of Laboratory Medicine, Second Medical Center of Chinese PLA General Hospital, National Clinical Research Center for Geriatric Diseases, Beijing, China; 2 Department of Laboratory Medicine, Fifth Medical Center, Chinese PLA General Hospital, Beijing, China

**Keywords:** 25(OH)D, PTH, Threshold, Reference interval, Determinant

## Abstract

**Purpose:**

By recruiting reference population, we aimed to (1): estimate the 25(OH)D threshold that maximally inhibits the PTH, which can be defined as the cutoff value for vitamin D sufficiency; (2) establish the PTH reference interval (RI) in population with sufficient vitamin D.

**Methods:**

Study data were retrieved from LIS (Laboratory Information Management System) under literature suggested criteria, and outliers were excluded using Tukey fence method. Locally weighted regression (LOESS) and segmented regression (SR) were conducted to estimate the threshold of 25(OH)D. Multivariate linear regression was performed to evaluate the associations between PTH concentration and variables including 25(OH)D, gender, age, estimated glomerular filtration rate (EGFR), body mass index (BMI), albumin-adjusted serum calcium (aCa), serum phosphate(P), serum magnesium(Mg), and blood collection season. *Z* test was adopted to evaluate whether the reference interval should be stratified by determinants such as age and gender.

**Results:**

A total of 64,979 apparently healthy subjects were recruited in this study, with median (Q1, Q3) 25(OH)D of 45.33 (36.15, 57.50) nmol/L and median (Q1, Q3) PTH of 42.19 (34.24, 52.20) ng/L. The segmented regression determined the 25(OH)D threshold of 55 nmol/L above which PTH would somewhat plateau and of 22 nmol/L below which PTH would rise steeply. Multivariate linear regression suggested that gender, EGFR, and BMI were independently associated with PTH concentrations. The PTH RI was calculated as 22.17–72.72 ng/L for subjects with 25(OH)D ≥ 55 nmol/L with no necessity of stratification according to gender, age, menopausal status nor season.

**Conclusion:**

This study reported 25(OH)D thresholds of vitamin D sufficiency at 55 nmol/L and vitamin D deficiency at 22 nmol/L, and consequently established PTH RIs in subjects with sufficient vitamin D for northern China population for the first time.

**Supplementary Information:**

The online version contains supplementary material available at 10.1007/s40618-023-02057-9.

## Introduction

Parathyroid hormone (PTH) and vitamin D play critical roles in regulation of mineral metabolism, as they form a tightly controlled feedback cycle in the maintenance of calcium, phosphate homeostasis, and bone health [1]. The serum 25-hydroxyvitamin D [25(OH)D] concentration is generally accepted as the best biomarker to determine the vitamin D status [2]. One well-known physiological function of 25(OH)D is to inhibit PTH in a moderate level, so as to avoid the adverse effect of elevated PTH concentration (secondary hyperparathyroidism). It is an attractive idea to estimate the 25(OH)D threshold (inflection or breaking point) for the maximal suppression of PTH, which is considered to be vitamin D sufficiency [2–6]. Numerous researches have reported the estimated thresholds ranged from 10 to 125 nmol/L [7, 8], while some studies failed to find a threshold [9, 10].

There has been considerable debate on the 25(OH)D threshold that defines vitamin D sufficient or optimal [2, 11], despite a large number of randomized clinical trials and evidence-based studies. Nevertheless, the exploring relationship between PTH and 25(OH)D which takes confounding factors into consideration is still a recommended approach for the foreseeable future [2].

On the other hand, a PTH measurement is essential in the diagnosis of calcium/phosphorus metabolism disorders, parathyroid dysfunction monitoring, and chronic kidney disease (CKD) patient care. An accurate PTH reference interval (RI), especially the upper reference limit (URL) determination, is fundamental in differential diagnosis of hyperparathyroidism (HPT) [12]. What’s more, the PTH URL also plays an important role in clinical management for patients with CKD. For example, in non-dialysis cases, the PTH concentrations are recommended within the normal range, while in dialysis patients, a higher PTH level (2 to ninefold of the URL) is suggested by guidelines [13]. However, it is not an easy task to establish the standard PTH RIs. The first step to establish PTH RIs is to recruit a reference population under critical criteria, any situation possibly affecting PTH concentration should be excluded [14]. It is strongly recommended that PTH RIs should be established among vitamin D sufficient subjects with a normal renal function and a normal serum calcium/ phosphate concentration [13–17]. Another issue concerning PTH RIs establishment is evaluation for possible stratification according to PTH determinants such as age, gender, and season [14, 18].

The elusive definition of vitamin D sufficiency, the complexity of reference population criteria, the debate concerning age and gender effects on PTH, and the variations in different countries and ethnicities collaboratively hampered attempts to get generally accepted PTH RIs. Accordingly, adequately powered studies to establish PTH RIs in Chinese population are still missing. The commonly used 25(OH)D cutoff values and PTH RI are provided by manufacturers’ instructions and are mostly unverified, which may lead to erroneous diagnoses and false medical decisions.

In this study, we first recruited a reference population. By evaluating the relationship between PTH and 25(OH)D and exploring the determinants of PTH, we aimed to estimate the 25(OH)D threshold that maximally inhibits the PTH and to report PTH RIs for total population and subjects with different 25(OH)D concentrations. Furthermore, we investigated whether the PTH RI partitions should be kept separate based on gender, age, menopausal status or season in vitamin D sufficient population with normal renal function.

## Materials and methods

### Study population

This is a cross-sectional study in subjects who had undergone routine physical examinations at the Health Management Institute of the Chinese PLA General Hospital from January 2014 to December 2019. We retrospectively retrieved data from subjects who had serum 25(OH)D and PTH tests simultaneously, and their corresponding serum biochemical parameters, gender, age, BMI, and date of measurement. All analytical data were extracted from the LIS (Laboratory Information Management System) of the Chinese PLA General Hospital. This study was approved by the Ethics Committee of Chinese PLA General Hospital (S2019-261-01).

### Inclusion and exclusion criteria

Data from patients with certain medical or medication records would not be included during data retrieval, including diagnosis of tumor, hyperparathyroidism, hypoparathyroidism, osteomalacia, and medications of bisphosphonates, estrogen, parathyroid hormone preparations, glucocorticoid. And then we selected apparently healthy subjects by evaluating their phosphocalcium metabolism, renal function, and liver function. Data were only included when individuals were aged over 18 years and the following serum biochemical parameters were within the respective reference intervals: aCa (2.10–2.60 mmol/L), P (0.80–1.60 mmol/L), Mg (0.60–1.40 mmol/L), EGFR (≥ 60 mL/min/1.73m^2^). Individuals with serum ALT or AST > 100 U/L, or serum PTH > 195 ng/L were excluded from the analysis as they might have potential liver disease or parathyroid dysfunction.

### Sample detection

Fasting blood was drawn from 8 a.m. to 10 a.m., and serum biochemical assay was completed on the same day. All 25(OH)D and PTH tests were conducted by the electrochemiluminescence assay on Cobas e601 analyzer (Roche Diagnostics, Switzerland). According to manufacturer’s instructions, the measuring range of 25(OH)D assay was 7.5–175 nmol/L, and the within-assay coefficient of variation (CV) was < 6.8% and the intermediate CV was < 13.1%. A method comparison had been conducted to cross-calibrate the results of immunoassay: 25(OH)D concentrations of 909 samples measured by the Cobas e601 assay (Ccobas) were compared with those by an LC–MS/MS assay(C_MS_) (Calibra Laboratory, Beijing, China), and the LC–MS/MS assay was aligned with NIST SRM 972a and accepted by CAP(College of American Pathologists). Then all immunoassay 25(OH)D concentrations were converted to LC–MS/MS equivalents following the equation: *C*_MS_ = 1.039*Ccobas + 0.497, *R*^2^ = 0.591 (Supplementary Fig. 1) before analysis. For the second-generation PTH assay, the functional sensitivity was 6.0 pg/mL, and the reference range was 15–65 ng/L. Other biochemical parameters were measured on Cobas c501 analyzer (Roche Diagnostics, Switzerland). Albumin-adjusted serum calcium was calculated by adding the factor 0.02 × (43-albumin concentration in g/L) to the measured serum calcium concentration [19]. EGFR was calculated using the CKD-EPI equation [20]. For all laboratory analyses, the analyzer performance was monitored daily, and a national external quality assessment (EQA) was conducted to ensure the assay quality.

### Definition and categorization

Blood collection time was divided into four groups: spring (March, April, and May), summer (June, July, and August), autumn (September, October, and November), and winter (December, January, and February). The BMI was categorized as: underweight (< 18.5 kg/m^2^), normal weight (18.5–24.9 kg/m^2^), overweight (25.0–29.9 kg/m^2^), and obese (≥ 30 kg/m^2^). For the analyses, subjects were stratified into five age groups (18–34, 35–44, 45–59, 60–69, ≥ 70 years), and four EGFR groups (≥ 120, 120–100, 100–80 and 80–60 mL/min/1.73m^2^). Because menopausal status of women was not recorded in our database, we classified women under 45 years as pre-menopausal and women over 55 years as post-menopausal, as it was reported that about 90% of Chinese women have menopause between the ages of 45 and 55 [21].

### Statistical analysis

To retrieve a “normal population”, logPTH outliers were discarded using the Tukey fence method [22, 23] before all analyses. Visual inspection of the histograms was applied for normality test, as formal normality tests were very sensitive to a deviation from normality for large tests numbers in this study. Statistical analysis results were expressed as percent (%) for categorical variables, mean ± SD for variables approximately meet a normal distribution, and as medians (Q1, Q3) when variables were non-normally distributed. Serum PTH and 25(OH)D concentrations were non-normally distributed and were compared by the Kruskal–Wallis analysis among different subgroups and the rank-based ANOVA was used for pairwise comparison. Locally weighted regression smooth scatter plot (LOESS) [24, 25] was adopted to describe the relationship between 25(OH)D and PTH, and to explore the potential threshold of 25(OH)D. Subsequently, the exact values of cut-points of 25(OH)D was determined by the segmented regression (SR) [26]. Multivariate linear regression was performed to evaluate the associations between PTH concentration and variables. In all regression analyses, PTH was the dependent variable, while 25(OH)D, gender, age, EGFR, BMI, aCa, P, Mg, and season were introduced as independent variables. To determine the PTH RI, the extreme percentiles (2.5 and 97.5) of serum PTH concentrations were computed overall and adjusted by 25(OH)D status. A Z test suggested by Harris [27] and Delgado [18] was adopted to evaluate whether the reference interval should be kept separate by determinants such as age, gender, menopausal status or season.

The LOESS model fitting and segmented regression was conducted by the R software (version 4.0.5). IBM SPSS Statistics 19 software (SPSS Inc., Chicago, IL, USA) was used for other statistical analyses. All figures were made by GraphPad Prism 8 (GraphPad Software Corporation, California, USA) and presented as mean ± SEM. For all analyses, *P* < 0.05 was considered statistically significant.

## Results

### Analysis population

**Fig. 1:**
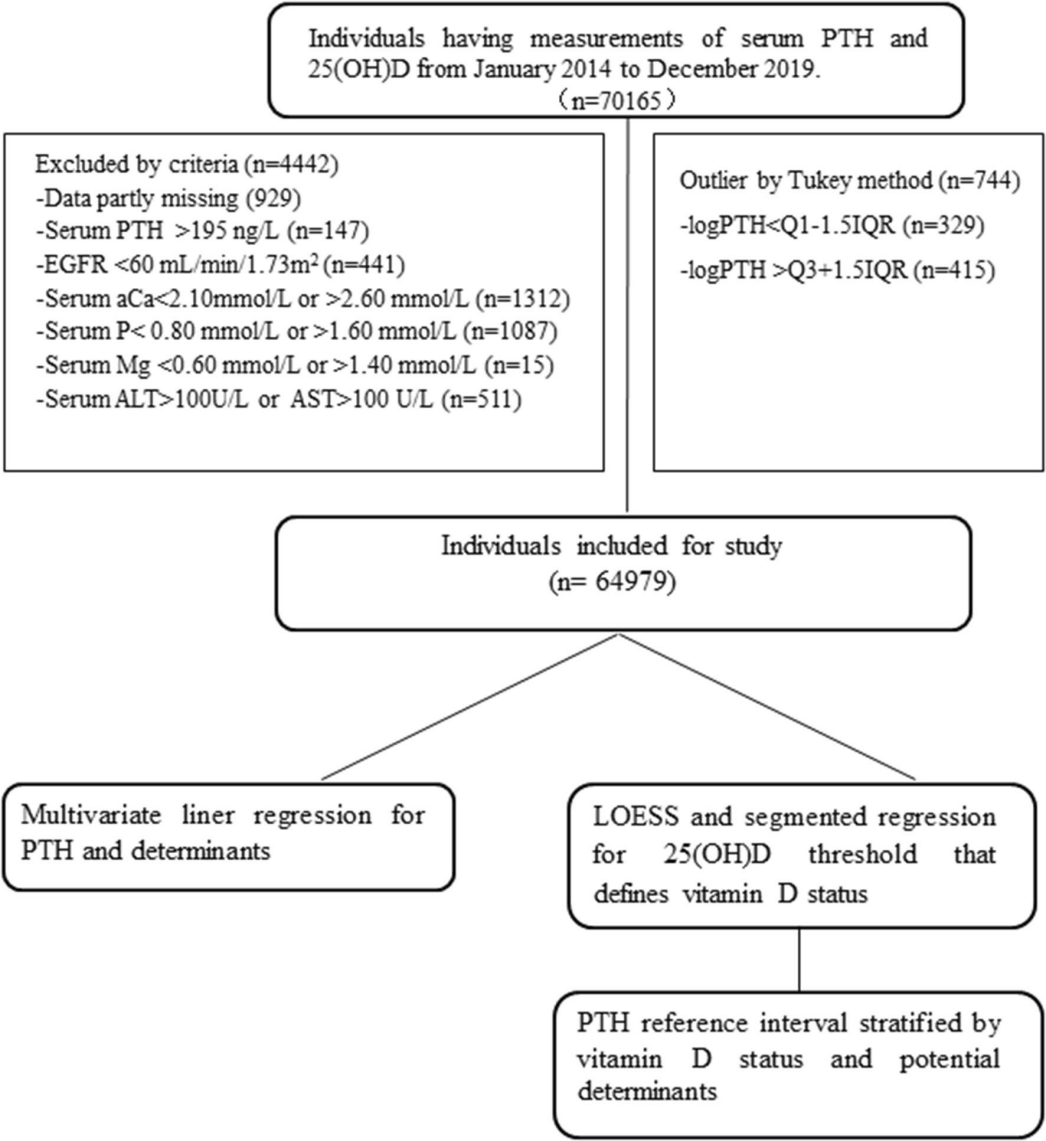
Study flow chart

Results from 64,979 individuals, among which 42,669 (65.67%) were men and 22,310 (34.33%) were women, aged 18–89 years (50.38 ± 9.46) were included in this study (Fig. 1). The 25(OH)D and PTH were non-normally distributed while the logPTH, aCa, P, Mg showed approximate normal distributions (Supplementary Fig. 2). The mean BMI of the subjects was 25.23 ± 3.41 kg/m^2^, serum aCa was 2.27 ± 0.08 mmol/L, serum P was 1.21 ± 0.16 mmol/L, and serum Mg was 0.89 ± 0.06 mmol/L. The population characteristics and PTH, 25(OH)D concentrations are described in Table 1.

**Table 1:** Descriptive statistics of the study population

	*n*	%	PTH ng/L Median (Q1,Q3)	*P*	25(OH)D nmol/L Median(Q1,Q3)	*P*
Total	64,979		42.19(34.24, 52.20)		45.33 (36.15, 57.50)	
Gender						
Man	42,669	65.67	42.08 (34.19, 51.85)	0.000	46.90 (38.08, 59.63)	0.000
Women	22,310	34.33	42.43 (34.32, 52.97)		42.18 (32.45, 52.78)	
Menopausal status						
Pre-menopausal	6324	–	44.85(34.35,52.95)	0.000	40.51(30.25, 47.98)	0.000
Post-menopausal	6947	–	43.34(35.07, 54.40)		45.00(36.15, 57.38)	
Age				0.000		0.000
18–29	1278	1.97	39.38 (31.53, 58.23)	a	44.21 (36.33, 53.61)	a
30–39	5544	8.53	42.42 (34.48, 52.43)	b	43.69 (35.40, 54.18)	a
40–49	23,054	35.48	42.95 (34.49, 52.54)	b	43.93 (34.38, 55.35)	a
50–59	25,631	39.45	41.88 (34.09, 51.73)	b	45.98 (37.03, 58.45)	b
60–69	7349	11.31	42.44 (34.16, 52.36)	b	48.50 (38.96, 62.53)	c
≥ 70	2123	3.27	43.76 (34.54, 56.23)	c	48.20 (37.50, 64.25)	c
Season				0.000		0.000
Spring	19,625	30.20	42.42 (34.26, 52.72)	a	41.93 (32.03, 52.60)	a
Summer	17,350	26.70	42.12 (34.27, 51.96)	b	48.60 (39.79, 61.38)	b
Autumn	15,576	23.97	40.87 (33.22, 50.35)	c	49.16 (39.93, 62.35)	c
Winter	12,428	19.13	43.63 (35.64, 54.07)	d	41.98 (32.96, 51.93)	d
BMI				0.000		0.000
< 18.5	1051	1.62	39.78 (32.60, 50.19)	a	43.20 (33.98, 54.34)	a
18.5–24.9	30,154	46.41	41.53(33.81, 51.30)	b	45.35(35.90, 58.13)	b
25–29.9	28,569	43.97	42.68(34.65, 52.85)	c	45.73(36.83, 57.65)	b
≥ 30	5205	8.01	44.60 (36.13, 55.74)	d	44.40 (36.14, 55.05)	a

For pairwise comparison among different subgroups, if there were significant differences between two subgroups (*P*< 0.05), they were marked by: a, b, c, d

### Relationship between 25(OH)D and PTH, and the threshold for vitamin D status

**Fig. 2:**
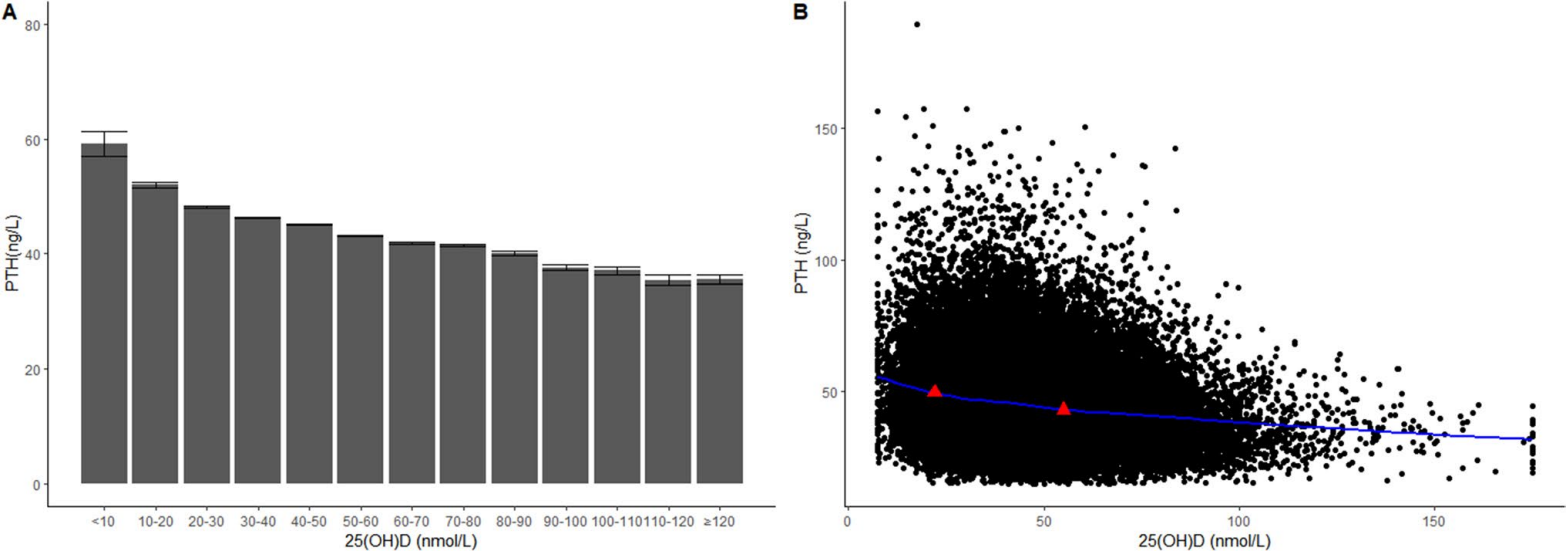
Serum PTH concentration according to 25(OH)D status (*n *= 64,979). **A** Histograms between 13 groups of different 25(OH)D concentrations and PTH concentrations. **B** Locally weighted regression smoothing (LOESS) curves between concentrations of 25(OH)D and PTH. Red triangles: cutoff values determined by segmented regression (22 nmol/L, 55 nmol/L)

To illustrate the relationship between PTH and 25(OH)D concentrations, we first stratified the 25(OH)D concentrations in 13 groups at an increment interval of 10 nmol/L, i.e., the first group was 25(OH)D < 10 nmol/L and the final group was 25(OH)D ≥ 120 nmol/L (Fig. 2A). As shown in Fig. 2A, there was a nonlinear negative correlation between serum 25(OH)D and PTH. Considering the complexity of the relationship, we adopted LOESS method to fit a smooth curve (Fig. 2B). Both the column chart and the LOESS curve visually suggested that there might be two 25(OH)D inflection points. Using segmented regression, it was suggested that PTH reached its plateau (decreased slightly) when 25(OH)D was 55 nmol/L or more, while PTH rose steeply when 25(OH)D was less than 22 nmol/L. Therefore, we defined vitamin D status as: sufficient (≥ 55 nmol/L), insufficient (22–55 nmol/L), and deficient (< 22 nmol/L). Multivariate linear regression coefficients of 25(OH)D in subjects with varied 25(OH)D concentrations are listed in Table 2, and weakened coefficient was observed in subjects with 25(OH)D sufficient.

**Table 2:** Multivariate linear regressions coefficients of 25(OH)D stratified by 25(OH)D concentrations

25(OH)D (nmol/L)	*n*	*R*^2^	Coefficient	Coefficient 95% CI	
Total	64,979	0.122	− 0.143	− 0.15	− 0.137
< 22	3274	0.132	− 0.341	− 0.494	− 0.188
22–55	42,770	0.107	− 0.156	− 0.173	− 0.14
< 55	46,044	0.113	− 0.181	− 0.195	− 0.168
≥ 55	18,935	0.095	− 0.097	− 0.111	− 0.083

### Determinants of PTH

**Fig. 3:**
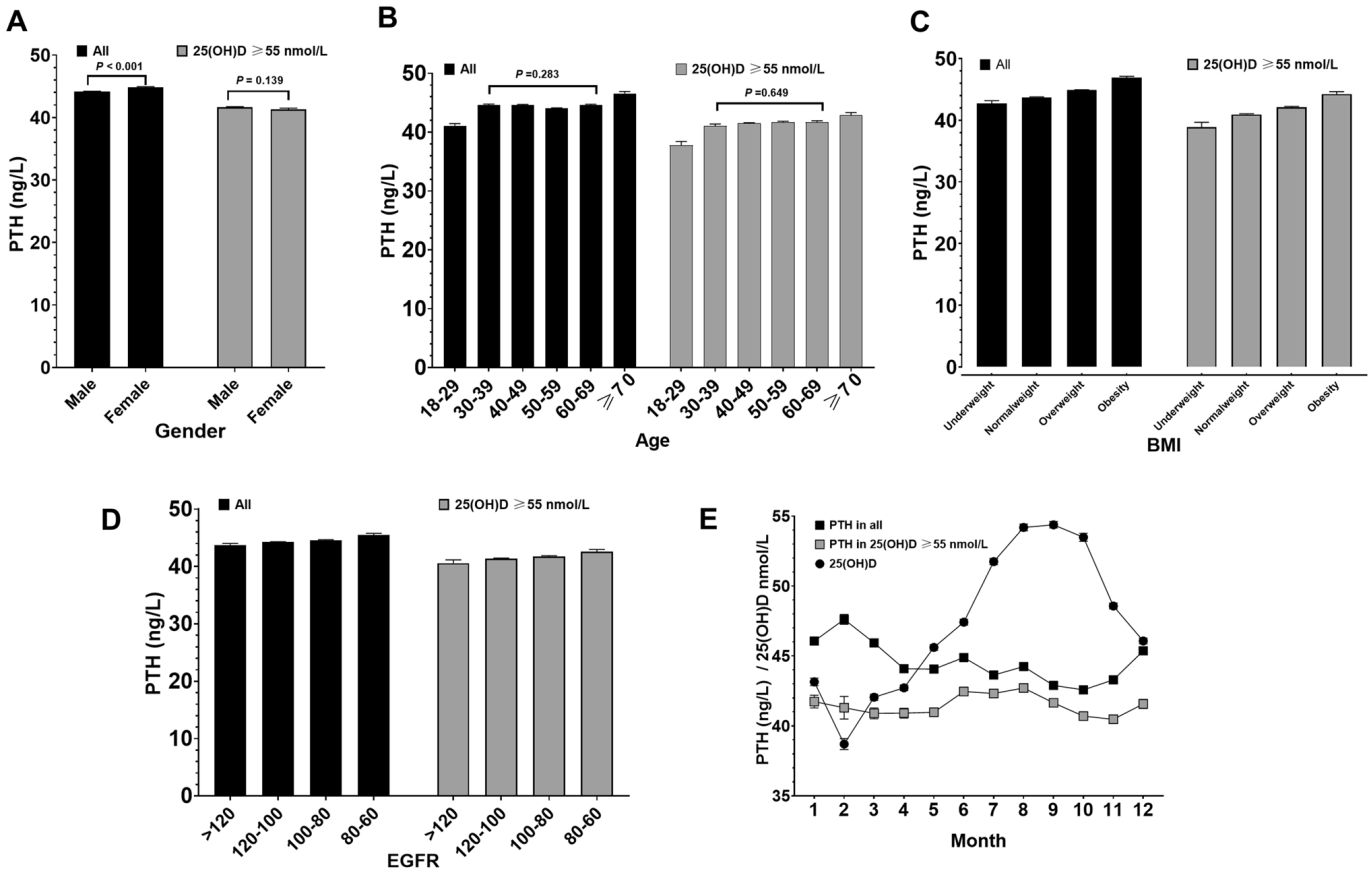
PTH concentrations in all subjects and in subjects vitamin D sufficient grouped by associated variables. PTH concentrations in different gender (**A**), age groups (**B**), BMI groups (**C**), EGFR groups (**D**) and PTH/25(OH)D concentrations in different months (**E**) among all population and population with 25(OH)D ≥ 55 nmol/L

PTH concentrations grouped by gender, age, BMI, EGFR, and blood collection months are shown in Fig. 3. In general, gender, age, BMI, EGFR, and seasonal variation affected PTH concentrations to various degrees. Overall, the PTH concentration of men was lower than that of women (*P* < 0.001), yet no difference was observed in subjects with 25(OH)D ≥ 55 nmol/L (*P* = 0.139) (Fig. 3A). No PTH concentration difference was observed among the four age groups 30–39, 40–49, 50–59, and 60–69 years (*P* = 0.283), while the lowest and highest PTH concentrations were observed in individuals aged 18–29 and  ≥ 70 years, respectively. A similar trend reappeared in subjects with 25(OH)D ≥ 55 nmol/L (Fig. 3B). We also observed ascending PTH concentrations with the increase of BMI (Fig. 3C) and the decrease of EGFR (Fig. 3D), regardless of the 25(OH)D concentrations. Figure 3E illustrated a seasonal variation effect on PTH concentrations: in total subjects, PTH concentrations (dark square) fluctuated inversely with the 25(OH)D concentrations (dark dot) throughout the year; however, the time-related PTH variation weakened and turned irregular among subjects with 25(OH)D ≥ 55 nmol/L (light square).

Multivariate linear regression in all subjects indicated that women, increased age, serum Mg, and BMI were associated with elevated PTH, but EGFR, serum P, aCa, 25(OH)D were negatively associated with PTH concentration (Table 3) after adjustment. However, in subjects with 25(OH)D ≥ 55 nmol/L, age was no longer a relevant factor affecting PTH concentration (*P* = 0.054).

**Table 3:** Multivariate linear regression for PTH in all subjects and in subjects with 25(OH)D ≥ 55 nmol/L

Variables	Total (*n* = 64,979, *R*^2^ = 0.122)				25(OH)D ≥ 55 nmol/L(*n* = 18,935, *R*^2^ = 0.095)			
	*P* value	Coefficient	Coefficient 95% CI		*P* value	Coefficient	Coefficient 95% CI	
Constant	0.000	110.16	105.741	114.579	0.000	92.889	85.526	100.252
Gender (vs. men)								
Women	0.000	2.779	2.529	3.029	0.000	2.501	2.041	2.961
Age	0.005	0.017	0.005	0.028	0.054	0.019	0.000	0.038
EGFR	0.000	− 0.055	− 0.067	− 0.043	0.010	− 0.033	− 0.053	− 0.014
P	0.000	− 20.643	− 21.354	− 19.932	0.000	− 18.571	− 19.799	− 17.343
aCa	0.000	− 25.913	− 27.371	− 24.456	0.000	− 20.502	− 22.911	− 18.093
Mg	0.000	20.449	18.729	22.169	0.000	17.842	14.985	20.700
25(OH)D	0.000	− 0.143	− 0.15	− 0.137	0.000	− 0.097	− 0.111	− 0.083
BMI	0.000	0.399	0.366	0.433	0.000	0.372	0.313	0.431
Season (vs. summer)								
Autumn	0.000	− 0.648	− 0.947	− 0.349	0.000	− 0.990	− 1.439	− 0.540
Winter	0.027	0.363	0.041	0.684	0.001	− 0.972	− 1.559	− 0.385
Spring	0.000	− 0.942	− 1.229	− 0.656	0.000	− 1.596	− 2.098	− 1.094

### PTH reference intervals

We investigated whether the PTH RI partitions should be kept separate based on gender, age, menopausal status or season in consideration of PTH determinants and clinical practice. Specifically, Z test was performed with gender and age (30, 70 years) as cutoff points based on our previous results (Fig. 2). *Z* test results (shown in Table 4) suggested that there was no need to stratify the PTH RIs based on gender, age, menopausal status, and season, neither in the general population nor in subjects with 25(OH)D ≥ 55 nmol/L. Finally, we established the PTH RIs in subjects with vitamin D sufficient, which was defined in this study. We also listed PTH RIs based on varied 25(OH)D values commonly recommended for vitamin D sufficiency for the clinical use(Table 5). PTH RIs grouped by gender, menopausal status, age, and season were adjusted for 25(OH)D concentrations and presented in supplementary Table 1 for reference.

**Table 4:** Z and Z* values in PTH RIs establishment

Subjects	Groups	Z	Z*	Intervals kept separate
Total	Men/women	9.14	70.35	No
	< 30y/ ≥ 30y	45.21	70.35	No
	< 70y/ ≥ 70y	27.17	70.35	No
	Pre/post-menopausal	7.09	29.43	No
	Summer–autumn/winter–spring	19.01	70.35	No
25(OH)D ≥ 55 nmol/L	Men/women	2.39	37.68	No
	< 30y/ ≥ 30y	31.27	37.68	No
	< 70y/ ≥ 70y	9.26	37.68	No
	Pre/post-menopausal	5.87	13.97	No
	Summer–autumn/winter–spring	4.50	37.68	No

According to Harris’s methodology, if z > z*, both groups should be kept separate

**Table 5:** Serum PTH reference interval adjusted for 25(OH)D concentration

		25(OH)D (nmol/L)				
		Total	≥ 30	≥ 50	≥ 55	≥ 75
*n* (%)		64,979	55,705 (85.73)	25,139 (38.69)	18,935 (29.14)	4465 (6.87)
PTH RIs (ng/L)	2.5th percentile	23.20	22.99	22.34	22.17	21.49
	97.5th percentile	78.58	76.98	73.24	72.72	68.51

## Discussion

To the best of our knowledge, this study was the first attempt exploring the 25(OH)D threshold and establishing the standard PTH RIs for the northern China population using big data covering all age groups. We established a reference population in strict accordance with the criteria recommended in literatures, and reasonably eliminated the subjects with potential factors which may affect the relationship between 25(OH)D and PTH. This is in agreement with relevant studies, where a normal renal/liver function [16] and normal phosphocalcium metabolism [11] were used in the inclusion criteria. In addition, a recent study revealed that an adequate magnesium concentration was likely required for vitamin D sufficiency [28]. Therefore, in this study, an abnormal serum magnesium concentration was set as one of the exclusion criterion.

By evaluating the relationship between 25(OH)D and PTH, we calculated a 25(OH)D threshold of ≥ 55 nmol/L for vitamin D sufficiency and a 25(OH)D threshold of < 22 nmol/L for vitamin D deficiency. Although currently, there has been no universally accepted 25(OH)D threshold for vitamin D status, the estimated 25(OH)D cutoff values in this study were close to those recommended by the IOM guidelines [29].

A J Sai et al. [8] reported that the 25(OH)D threshold for PTH suppression varied from 25 to 125 nmol/L in a literature review of 70 studies. The considerable variability in the reported 25(OH)D thresholds can be explained by the methodology variations in 25(OH)D assays, the inconsistent statistical methods adopted for the threshold determination, and the varied study population in terms of ethnicity, age, diet and culture which affect the relationship between 25(OH)D and PTH. Moreover, the obscure definition for the concept of threshold is also an unneglectable factor. To be specific, using different statistical models, some studies described the threshold as the breakpoint of 25(OH)D concentration below which serum PTH rises steeply, and some studies focused on the breakpoint above which serum PTH would somewhat plateau or decrease slightly. Other studies also reported the threshold as a 25(OH)D concentration region between the two breakpoints mentioned above.

A study in the USA revealed that the relationship between 25(OH)D and PTH varied with respect to race/ethnicity [30]. With respect to East Asian countries, a study in Japan [31] reported a 25(OH)D threshold of 50–75 nmol/L using the Diasorin 25(OH)D assay, and two studies from South Korea [32, 33] reported the threshold as 50 nmol/L using liquid chromatography–tandem mass spectrometry (LC–MS/MS) for 25(OH)D detection and 53 nmol/L for men, 34.5 nmol/L for women using Abbott 25(OH)D assay, respectively. The threshold in the present study was lower than that reported in Japan and slightly higher than that in South Korea. Besides the potential race-related variation in interpreting the study results, it must be noted that the 25(OH)D assays in the studies differed. It was well recorded that 25(OH)D immunoassays of different manufactures performed highly variable, and even LC–MS/MS methods did not necessarily produce comparable results [34]. In China, only a few small-scale studies have reported similar thresholds. A study on middle-aged and elderly in Shanghai, China reported that PTH was maximally suppressed at 25(OH)D concentration of 55 nmol/L (*n* = 1829) [7]. Another study in Chinese women of childbearing age (*n* = 1829) reported a 25(OH)D threshold of 53.7 nmol/L [35]. Both of the studies estimated similar 25(OH)D threshold to our study, using the same 25(OH)D assay(Roche Cobase601) and statistical model (LOESS) in calculating the threshold. However, one study in China reported a considerably lower threshold of 45 nmol/L [*n* = 1436, Roche Cobase601 for 25(OH)D assay] [36]. We inferred that the differed thresholds were relevant to the variations in statistical models and the study population criteria adopted. These studies seem to indicate that with standardized assay and proper statistical method, we are approaching the urgently needed 25(OH)D threshold for China population, which is critical for informing public health recommendations.

Besides the evaluation of the 25(OH)D and PTH relationship, our study also explored the effects on serum PTH concentrations of gender, age, EGFR, BMI, season and serum aCa, P, Mg. In our study, men had lower PTH than women, in almost all age groups (data not shown). This can be explained by the higher 25(OH)D concentrations in men recorded by most studies in Asia [37]. Regular effects of BMI and EGFR were observed in this study: PTH concentrations rose gradually with the increase of BMI (Fig. 3C) or the decline of EGFR (Fig. 3D). Similarly, multivariate linear regression showed that BMI (*β* = 0.399/0.372) and EGFR (*β* = − 0.055/− 0.033) independently associated with PTH, regardless of 25(OH)D concentrations. Being consistent with prior studies [16, 38], the results suggest that special attention should be paid to patients with CKD and obesity in the interpretation of PTH results. One hypothesis to explain the positive correlation between PTH and BMI in vitamin D sufficient population is that sequestration of vitamin D in the adipose tissue occurs, leading to a decreasing bioavailability of vitamin D [39]. In addition, 1,25(OH)_2_D feeds back to inhibit PTH secretion indirectly through its calcemic action, as well as by exerting a direct inhibitory action on PTH biosynthesis and parathyroid cell proliferation. It has been reported a tendency to a decreased expression of the 1-α hydroxylase and lower 1,25(OH)_2_D in cases of obesity [40]. Although the highest PTH concentration was observed in the age group of ≥ 70 years, our study did not support that merely increasing age can cause PTH to rise. In contrast, following observations suggest that age may not be an independent factor of PTH increase: (1) no significant increase of PTH was observed in the age ranged 30–70 years old (Fig. 3B); (2) in subjects with 25(OH)D ≥ 55 nmol/L, age was no longer a relevant factor affecting PTH concentrations (*P* = 0.054) (Table 3). We assumed that higher PTH concentrations observed in older people may be caused by accumulated age-related effects such as the declining EGFR and 25(OH)D concentrations, rather than age itself. This was supported by Rejnmark [41], but differed from the study of Touvier [38] and Dan [16]. Concerning the seasonal influence, the PTH concentrations in the overall population showed a sinusoidal-like change across a year, and accordingly the PTH concentrations in summer and autumn were lower than that in spring and winter. This agrees with many others studies [42, 43], and can be explained by the seasonal change of 25(OH)D concentrations. It is notable that the time-related PTH variation weakened and turned irregular when vitamin D was sufficient (≥ 55 nmol/L), and the maximum monthly change of PTH concentration was only 2.24 ng/L (vs. 5.04 ng/L in total population) (Fig. 3E). This seems to suggest that a 25(OH)D concentration above 55nmo/L was enough to suppress PTH within slight change.

Gender, age, menopausal status, and season were the mostly discussed potential factors for PTH RIs stratification. In this study, they showed limited influence on PTH concentrations, which was not strong enough to determine PTH RI stratifications. Recent studies suggested that it was not necessary for gender-adjusted PTH RI partitions, which was in line with our study, despite the statistically higher PTH concentration observed in women [18, 44]. Although there is still controversy about whether PTH RI should be stratified according to age [14, 18, 19, 38, 41], most studies have reported that elderlies have higher PTH concentrations than middle-aged individuals. We believe that a large sample covering all age groups and appropriate statistical criteria are necessary to obtain credible conclusion on this issue.

We reported a PTH RI of 23.20–78.58 ng/L for the total population and 22.17–72.72 ng/L for subjects with 25(OH)D ≥ 55 nmol/L, and the URL of which were 20.89% and 11.88% higher than the manufacturer’s instructions (Roche, 15–65 ng/L), respectively. This could be relevant to the fact that manufacturers tend to recruit young donors for the reference population [45]. In addition, we calculated a PTH RI of 20.23–64.62 ng/L in subjects ≤ 40 years with 25(OH)D ≥ 75 nmol/L, where the URL of which was consistent with the manufacturer’s recommendation. Consequently, we speculated that Roche applied the 25(OH)D cutoff value of 75 nmol/L for vitamin D sufficiency in establishing PTH RIs. It is easy to understand that the younger age and higher 25(OH)D concentrations of the reference population used by the manufacturer may cause a super-healthy cohort with an unnaturally narrow range of PTH results. Similar to our work, two studies in the establishment of PTH RIs using data mining method have reported a considerably higher URL than the manufacturer’s recommendation [18, 19].

This study has several strengths. The sample size was relatively large to provide more statistical power in evaluating the relationship between 25(OH)D and to examine related variables comprehensively. The study data were retrieved from subjects undergoing routine physical examinations under strict criteria rather than patients, so the ineligible individuals were excluded as possible. For instance, we tentatively introduced serum Mg as an inclusion/exclusion criterion from recent literature, and we saw a strong association of PTH with Mg in multiple linear regression. We, for the first time, reported the 25(OH)D threshold for vitamin D sufficiency and consequent PTH RIs in the northern China population. Furthermore, we demonstrated that the newly reported 25(OH)D threshold (55nmoll/L) may be more proper than the cutoff value of 75 nmol/L recommended by the manufacture. Although this is a very large cross-sectional study, we acknowledge several limitations. The PTH RIs are only applicable to the Roche immunoassays. This is the inherent limitation of PTH methodology: dramatically different performances of varied PTH assays have been recorded. And the results of this study are not automatically applicable to other populations as varied vitamin D bioavailability in different ethnic groups has been reported. Another important limitation is that calcium intake data were unavailable in this retrospective study. It has been reported that PTH increases in case of chronic low calcium intake [14]. Mathilde Touvier et al. [38] reported that in subjects with insufficient 25(OH)D concentration, the 97.5th percentile for PTH was higher in those with lower calcium intake, but this difference disappeared in those 25(OH)D sufficient. To minimize the impact of missing calcium intake data: (a) we excluded individuals with abnormal serum phosphate or renal function, which may affect the absorption and regulation of calcium; (b) only subjects with normal albumin-adjusted serum calcium (measured on the same sample than PTH) were included in this study; (c) we established PTH RIs in subjects with sufficient vitamin D. What’s more, the lack of vitamin D supplementation data may potentially weaken our findings. Considering that the use of vitamin D supplements is not common in China, and vitamin D supplement use is not a clear exclusion criterion in similar study focusing on the 25(OH)D threshold that maximally inhibits PTH, we think the bias brought by the lack of vitamin D supplementation data is acceptable.

In conclusion, this study found a 25(OH)D threshold for vitamin D sufficiency and established PTH RIs in apparently healthy subjects with sufficient vitamin D for the northern China population for the first time. Our data suggest that stratifications for PTH RIs according to gender and age are not necessary. In addition, we demonstrated that gender, EGFR, and BMI were independent factors for PTH concentrations, but age may not.

### Supplementary Information

Below is the link to the electronic supplementary material.

Supplementary file1 (DOCX 391 KB)

## Data Availability

Data not available due to ethical restrictions.
