# Supplementary material for: Threshold of 25(OH)D and consequently adjusted parathyroid hormone reference intervals: data mining for relationship between vitamin D and parathyroid hormone
Source: J Endocrinol Invest. 2023 Mar 15;46(10):2067–77. doi: 10.1007/s40618-023-02057-9 (PMC10514164; doi:10.1007/s40618-023-02057-9)
Supplement: Supplementary file 1 — Supplementary file1 (DOCX 391 KB) [file 40618_2023_2057_MOESM1_ESM.docx]

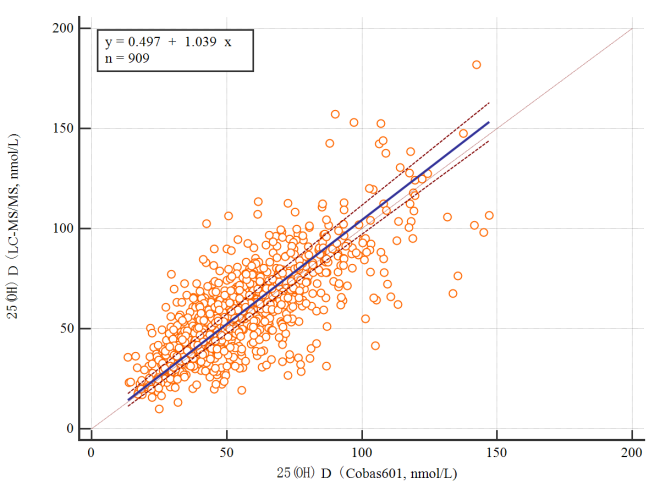


**Supplementary Figure 1 Passing-Bablok regression of 25(OH)D concentrations measured by Roche Cobas601 and LC-MS/MS with n=909 and R^2^=0.591.**

Serum samples of 909 healthy adults aged 37-96 years were collected in this method comparison study. The concentration of 25(OH)D levels ranged from 9.78 nmol/L to 181.95 nmol/L for LC-MS/MS. The LC-MS/MS assay for the concentration of 25(OH)D levels was conducted at Calibra Laboratory, Beijing, China. The LC–MS/MS method was aligned with NIST SRM 972a and accepted by CAP as a proficiency testing scheme for 25(OH)D assay.


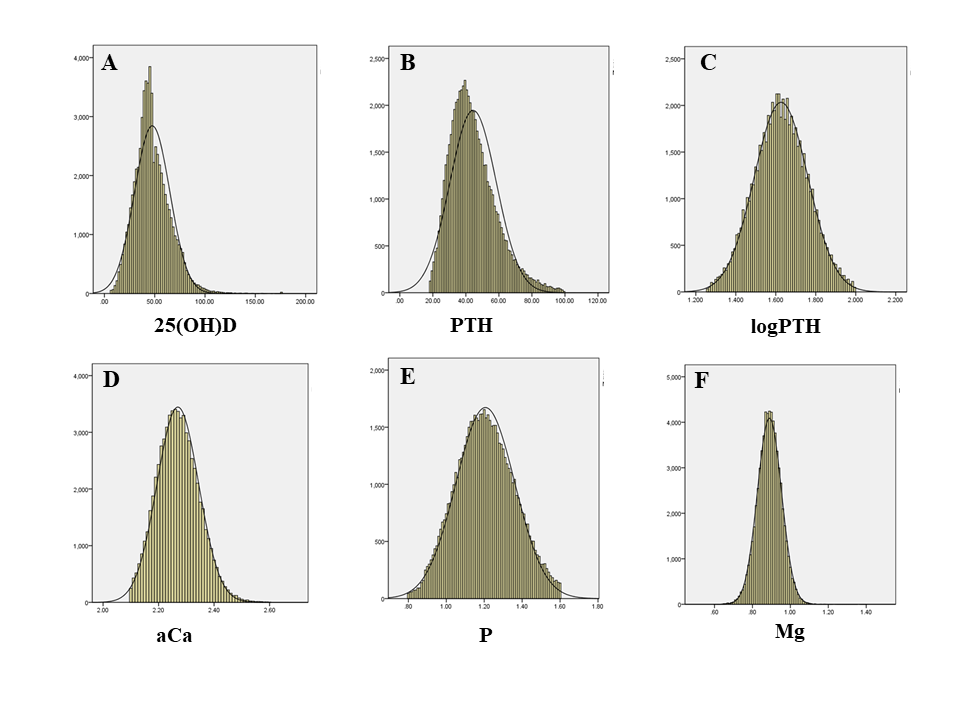


**Supplementary Figure 2 Histograms for serum biochemical variables (n=64979).**

25(OH)D (A), PTH (B) were non-normally distributed. LogPTH (C), albumin-adjusted calcium (aCa) (D), phosphate (P) (E) and magnesium (Mg) (F) showed approximate normal distributions.

| **Supplementary Table 1 Grouped PTH RIs adjusted for 25(OH)D concentration** | | | | | |
| --- | --- | --- | --- | --- | --- |
| **Groups** | **25(OH)D (nmol/L)** | | | | |
|  | **All concentrations** | **≥30** | **≥50** | **≥55** | **≥75** |
| **Total** | 23.18~78.58 | 22.98~76.98 | 22.32~73.24 | 22.15~72.72 | 21.45~68.51 |
| **Gender** |  |  |  |  |  |
| man | 23.03~77.36 | 22.85~75.96 | 22.31~72.88 | 22.08~72.34 | 21.44~68.44 |
| women | 23.41~80.45 | 23.22~78.87 | 22.33~74.54 | 22.31~73.60 | 21.51~69.04 |
| **Menopausal status** |  |  |  |  |  |
| pre-menopausal | 23.41~79.31 | 23.23~77.46 | 22.05~70.91 | 21.94~71.00 | 21.97~64.21 |
| post-menopausal | 23.51~82.74 | 23.34~81.57 | 22.55~78.03 | 22.57~76.40 | 20.91~71.12 |
| **Age** |  |  |  |  |  |
| ≤30 y | 21.98~72.97 | 21.97~71.83 | 20.92~63.73 | 20.99~62.20 | 19.80~64.26 |
| 31~69 y | 23.25~78.38 | 23.04~76.80 | 22.42~72.87 | 22.31~72.33 | 21.78~67.94 |
| ≥70 y | 22.34~88.45 | 22.04~82.92 | 20.91~82.45 | 20.92~79.31 | 19.68~75.81 |
| **Season** |  |  |  |  |  |
| spring | 23.22~79.22 | 22.88~77.41 | 21.95~73.74 | 21.97~72.37 | 21.33~66.38 |
| summer | 23.27~77.59 | 23.21~77.10 | 22.81~73.90 | 22.73~73.71 | 21.91~72.93 |
| autumn | 22.47~75.85 | 22.40~75.23 | 21.97~72.65 | 21.79~71.26 | 21.37~67.30 |
| winter | 24.13~80.80 | 23.82~78.32 | 22.74~71.78 | 22.35~71.64 | 21.35~64.38 |
